# Management and Prognostic Implications of Posteriorly Advanced OSCC in Contact With the Medial Pterygoid Muscle

**DOI:** 10.1002/cam4.71174

**Published:** 2025-11-20

**Authors:** Nanako Ito, Fumitaka Obayashi, Mirai Higaki, Atsuko Hamada, Sachiko Yamasaki, Tomoaki Shintani, Toshinori Ando, Koichi Koizumi, Souichi Yanamoto

**Affiliations:** ^1^ Department of Oral Oncology Graduate School of Biomedical and Health Sciences, Hiroshima University Hiroshima Japan; ^2^ Center of Oral Clinical Examination Hiroshima University Hospital Hiroshima Japan

**Keywords:** compartmental surgery, masticator space, medial masticator space dissection, oral squamous cell carcinoma, postoperative radiotherapy (PORT), recurrence, survival

## Abstract

**Background:**

T4b was previously defined as unresectable; however, it has been demonstrated that compartmental surgery can achieve treatment outcomes comparable to those of T4a cases. We frequently encounter posteriorly advanced oral squamous cell carcinoma (OSCC) in contact with the medial pterygoid muscle, and determining the appropriate extent of resection and managing subsequent recurrence in the masticator space remain challenging. Therefore, this study aimed to investigate the association between the tumor's spatial relationship with the medial pterygoid muscle and patient outcomes, providing insights to enhance diagnostic precision and guide therapeutic strategies.

**Methods:**

This retrospective study included 50 patients with posteriorly advanced OSCC, excluding those classified as T4b. Preoperative magnetic resonance imaging classified lesions as either non‐contact or contact type based on their relationship to the medial pterygoid muscle. Clinicopathological factors, overall survival rate (OS), and recurrence rates in the masticator space were compared between the two groups.

**Results:**

No significant differences were observed in OS between the non‐contact and contacttypes. Among non‐contact types, no recurrence in the masticator space was observed, whereas contact‐type tumors showed a recurrence rate of 22.6% (*p* = 0.04). Multivariate analysis revealed that lymphatic invasion was associated with an increased risk of primary recurrence, whereas postoperative radiotherapy was associated with a reduced risk among 31 patients with contact‐type tumors.

**Conclusions:**

In summary, compartmental surgery with pterygomandibular space dissection should be considered for contact‐type tumors near the medial pterygoid muscle, along with T4b cases. However, its indication must be assessed based on clinical findings. Postoperative radiotherapy is key to recurrence prevention, improving oncologic outcomes and long‐term disease control.

## 1 Introduction

Oral squamous cell carcinoma (OSCC) is the 13th most common cancer worldwide [1]. It is more widespread in developing countries compared to developed nations. However, due to lifestyle changes, this trend has recently shifted, with an increasing number of OSCC cases being reported in developed countries as well [2]. The standard treatment for patients with early‐stage OSCC involves surgery, whereas advanced cases with a high risk of recurrence require postoperative radiotherapy (PORT), with or without chemotherapy [3, 4]. For instance, locally advanced OSCC invading the masticator space is associated with poor survival outcomes [5], making it crucial to prevent recurrence. According to the 8th edition of the American Joint Committee on Cancer Staging Manual [6], tumors invading the masticator space—including the muscles of mastication (masseter, temporalis, medial, and lateral pterygoids), pterygoid plates, or skull base—and/or encasing the internal carotid artery are classified as T4b [7]. T4b has traditionally been considered unresectable. However, recent studies have demonstrated significant advancements in surgical treatment for T4b OSCC, suggesting that surgery may be a curative treatment option for selected patients. The masticatory space is classified into infra‐notch and supra‐notch regions based on the mandibular notch. Infra‐notch T4b tumors had survival rates similar to T4a, while supra‐notch T4b tumors had worse outcomes [8]. Additionally, Pillai et al. reported that lateral pterygoid muscle involvement was associated with poor prognosis [9]. According to a recent meta‐analysis, no significant difference in survival and local control between infra‐notch T4b and T4a OSCC was demonstrated [10].

Recent advancements in the study of OSCC invading the masticator space have led to the establishment of *compartment surgery*—a surgical approach that involves en bloc resection of the tumor together with the entire anatomical subunit it occupies—as an effective treatment strategy [11, 12]. However, the prognosis and optimal treatment approach for OSCC in contact with the masticator space—specifically, tumors in contact with the medial pterygoid muscle—remains unclear.

In such cases, a critical question arises: Is compartment surgery truly necessary, or is a conventional surgical approach sufficient? Despite its clinical significance, there is a lack of solid evidence to address this issue.

Therefore, in this study, we analyzed the spatial relationship between OSCC and the medial pterygoid muscle using preoperative magnetic resonance imaging (MRI) and evaluated its impact on surgical outcomes, including overall survival (OS), local recurrence rates, recurrence sites, potential prognostic factors for local control, and the necessity of pterygomandibular space dissection or postoperative radiotherapy (PORT) in posteriorly advanced OSCC.

This study aims to provide critical insights into the optimal surgical strategy for OSCC in contact with the medial pterygoid muscle, ultimately contributing to more effective treatment decision‐making.

## 2 Materials and Methods

### 2.1 Study Patients

Between December 2013 and December 2022, 216 patients with locally advanced OSCC who were treated at the Department of Oral and Maxillofacial Surgery at Hiroshima University Hospital were enrolled in the study. The inclusion criteria were as follows: posteriorly advanced OSCC, defined as OSCC extending posteriorly beyond the line connecting the maxillary and mandibular first molars [11]; availability of clinical and prognostic data; having undergone surgical treatment; histopathological confirmation of OSCC; and a minimum of six months of postoperative follow‐up. Patients with a diagnosis of T4b OSCC were excluded.

**FIGURE 1:**
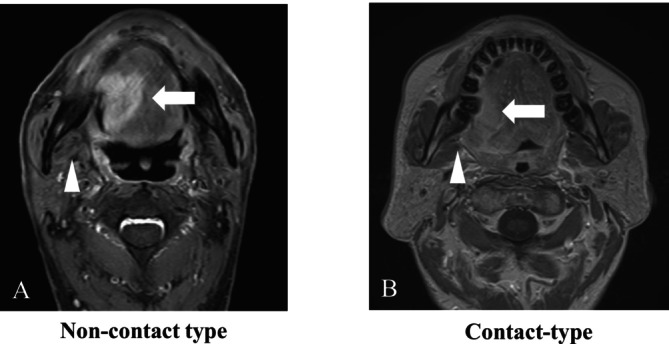
Classification by the position of posteriorly extended tumors relative to the medial pterygoid muscle.

Preoperative MRI scans were independently reviewed by two board‐certified oral and maxillofacial surgeons. Patients were classified into two groups based on the relationship between the posteriorly extended tumor and the medial pterygoid muscle (Figure 1): *Non‐contact type*: MRI findings demonstrated a distinct layer of adipose tissue interposed between the tumor and the medial pterygoid muscle, similar to the contralateral side. *Contact type*: MRI findings showed either complete loss or a marked reduction in the adipose tissue between the tumor and the medial pterygoid muscle compared to the contralateral side.

**FIGURE 2:**
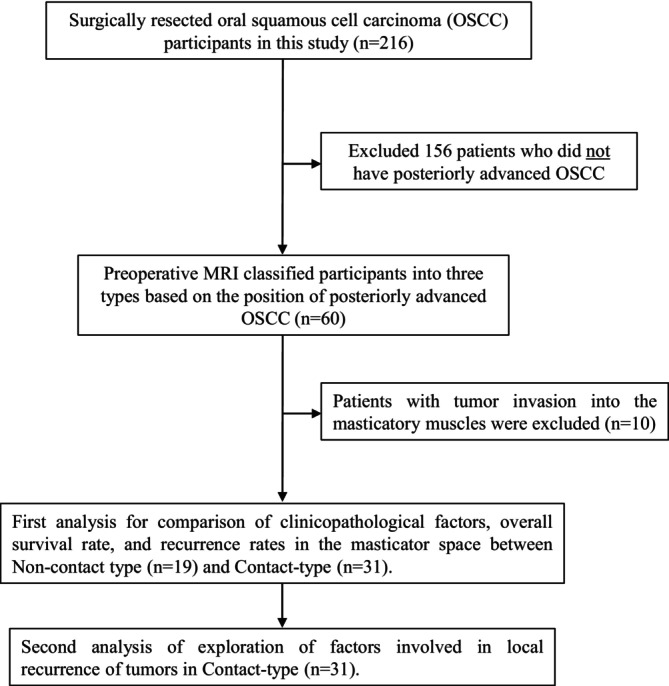
The flow diagram to participants for analysis in the study.

We first conducted an analysis comparing clinicopathological factors, overall survival rates, and recurrence rates in the masticator space between the non‐contact and contact types. Next, we investigated factors associated with local recurrence in the contact type (Figure 2).

### 2.2 Determination of Characteristic Variables of the Patients

Information on age, sex, performance status, primary site, clinical T stage, and pathological lymph node metastasis was obtained from the medical charts to identify potential factors influencing OSCC. Clinical data also included demographic details, pathological T and N stages, histological differentiation (categorized as well, moderately, or poorly differentiated), perineural invasion, vascular invasion, and lymphatic invasion. The primary evaluated endpoint was OS. Survival time was calculated from the date of surgery.

### 2.3 Statistical Analysis

All statistical analyses were performed using JMP 16 statistical software (SAS Institute Inc., Cary, NC, USA). Categorical variables were analyzed using the chi‐square test, as appropriate. Cumulative OS was estimated using the Kaplan–Meier method and compared using the log‐rank test. Cox proportional hazards analysis was used to explore factors involved in the local recurrence of the tumor. A two‐tailed *p* value of < 0.05 was considered statistically significant.

### 2.4 Ethical Considerations

This retrospective observational study was approved by the Institutional Review Board of Hiroshima University Hospital (approval number: epidemiology‐2023‐0025). The study protocol was made publicly accessible, allowing patients to opt‐out if they did not wish to participate. The requirement for informed consent was waived for this study.

## 3 Results

A total of 50 patients with posteriorly advanced OSCC were included in this study, comprising 19 (38.0%) females with a median age of 70.5 years (Table 1). Based on MRI classification, 19 (38.0%) patients were categorized as the non‐contact type and 31 (62.0%) as the contact type (Table 1). Regarding performance status, a significantly higher number of patients had a status of 0–1 compared to 2 or more (*p* < 0.05). The subsite of the primary cancer was the mandibular gingiva in most patients (*n* = 25), followed by the tongue (*n* = 12) and the maxillary gingiva (*n* = 7). Six cases received neoadjuvant chemotherapy. Although advanced T stage (T3/4) is considered a potential risk factor for recurrence, PORT was not routinely administered based solely on T stage. In our institution, PORT was recommended only when additional clinicopathological risk factors—such as positive margins, extranodal extension, or vascular invasion—were present.

**TABLE 1:** Clinicopathological characteristics of the study patients in non‐contact and contact‐type OSCC.

Variables	Total	Non‐contact	Contact
	(*n* = 50)	(*n* = 19)	(*n* = 31)
Sex			
Male	31	11	20
Female	19	8	11
Age	21–92	21–92	48–84
	(median 70.5)	(median 70.5)	(median 71.5)
Perfomance status			
0	35	10	25
1	10	4	6
2	2	2	0
3	2	2	0
4	1	1	0
Primary site			
Maxillary gingiva	7	4	3
Mandibular gingiva	25	15	10
Tongue	12	6	6
Buccal mucosa	6	0	6
Clinical T factor^a^			
1	1	1	0
2	15	9	6
3	10	5	5
4a	24	4	20
Pathological lymph node metastasis			
+	21	5	16
−	29	14	15
Preoperative treatment			
+	6	2	4
−	44	17	27
Postoperative radiotherapy (PORT)			
+	17	3	14
−	33	16	17
Surgical margin			
Positive	11	1	10
Close	8	2	6
Free	31	16	15
Histological grade			
Well	26	9	17
Moderately	21	9	12
Poor	3	1	2

^a^ TNM classification of the International Union for Cancer Control (UICC), 8th edition.

A certain portion of patients did not receive PORT for several reasons, among which the patients' will, surgeons' preference, and tolerance to postoperative radiotherapy are still the main reasons. Seventeen underwent PORT, including chemoradiotherapy (Table 1). In this study, compartmental surgery was not performed for any of the tumors contacting the medial pterygoid muscle. All patients underwent conventional tumor resection without anatomical subunit‐based dissection.

**FIGURE 3:**
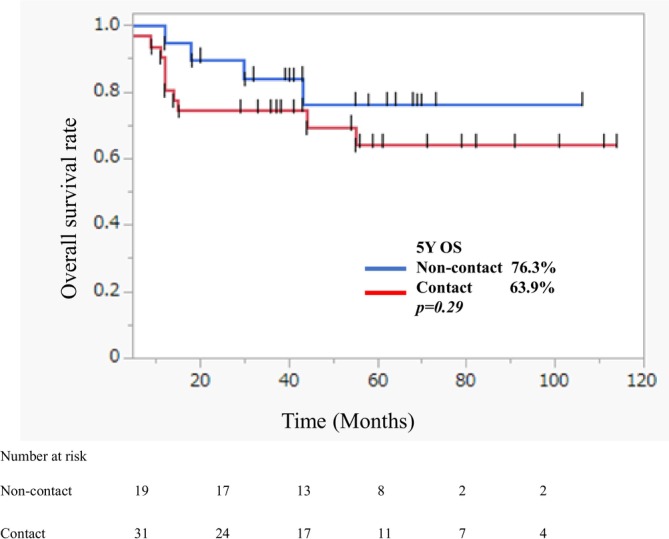
Kaplan–Meier curves of overall survival of the non‐contact and contact types of posteriorly advanced OSCC.

Although no significant difference in OS was observed between the non‐contact and contact types (Figure 3), the contact type showed a trend toward lower OS.

Of the 50 patients included in the study, 17 (34.0%) experienced local recurrence (Table 2). Seven of these cases were classified as the non‐contact type, and 10 as the contact type. Regarding the sites of local recurrence, recurrence in the mucosal surface layer was non‐contact type in six cases and contact type in three. Recurrence in the masticator space was not observed in the non‐contact type; whereas it occurred in seven (22.6%) cases in the contact type, a difference that was statistically significant (*p* = 0.04).

**TABLE 2:** Sites of local recurrence in non‐contact and contact‐type OSCC local recurrences occurred in 17 of 50 patients (34.0%).

Sites of recurrence	Non‐contact	Contact	*p* ^a^
	*n* = 7 (36.8%)	*n* = 10 (32.3%)	
Mucosal surface	6	3	0.07
Masticator space	0	7	0.04
Para‐hyoid area	1	0	0.38

^a^ Fisher's Exact Test: significant at *p* < 0.05.

Next, we explored the factors involved in the local recurrence of tumors in contact type. Univariate analysis identified PORT and lymphatic invasion as significant factors associated with recurrence in the masticator space. Multivariate analysis confirmed that both factors were independently associated with this type of recurrence. PORT was associated with a hazard ratio (HR) of 0.23 (95% confidence interval [CI], 0.12–0.36; *p* = 0.01); whereas lymphatic invasion had an HR of 12 (95% CI, 1.20–2.93; *p* = 0.03) (Table 3).

**TABLE 3:** Univariate and multivariate analysis of factors associated with primary site recurrence in contact‐type OSCC.

Variables	Univariate	Multivariate		
	*p*	HR	95% CI	*p* ^a^
Sex (male/famale)	1.00			
Age (≤ 67/> 67)	1.00			
Perfomance status (+/−)	0.36			
Clinical T factor^a^ (T4/T2, T3)	0.99			
Preoperative treatment (+/−)	0.28			
Postoperative radiotherapy (+/−)	< 0.01	0.23	0.12–0.36	< 0.01
Surgical margin (positive, close/free)	1.00			
Histological grade (well, moderately/poor)	0.67			
Perineural invasion (+/−)	1.00			
Lymphatic invasion (+/−)	0.01	12	1.20–2.93	0.03
Vascular invasion (+/−)	0.15			
Fat layer interposition (+/−)	0.15			
Medial pterygoid muscle resection (+/−)	0.29	4.98	0–5.56	0.22

Abbreviations: CI, confidence interval; HR, hazard ratio.

^a^ Cox regression analysis: significant at *p* < 0.05.

## 4 Discussion

Managing T4b OSCC presents significant challenges due to its advanced stage and frequent invasion of critical anatomical structures, such as the masticator space. This study revealed that contact‐type OSCC, characterized by tumors in direct contact with the medial pterygoid muscle, demonstrated a significantly higher recurrence rate in the masticator space compared to the non‐contact type. Importantly, lymphatic invasion and PORT were identified as independent factors contributing to masticator space recurrence in contact‐type tumors. These findings emphasize the necessity of developing revised treatment strategies to reduce recurrence rates in contact‐type OSCC.

### 4.1 Role of Lymphatic Invasion

Lymphatic invasion is a well‐established prognostic factor in head and neck cancers, indicating aggressive tumor behavior and an increased risk of both local and distant metastases [12, 13]. For contact‐type OSCC, lymphatic invasion likely facilitates tumor spread into the masticator space, increasing the risk of recurrence. Comprehensive lymph node dissection and meticulous pathological evaluation are critical to minimizing the risk of residual disease in these cases. Furthermore, the integration of intraoperative frozen section analysis and advanced molecular diagnostic techniques may assist in identifying microscopic lymphatic invasion and optimizing surgical strategies.

### 4.2 Postoperative Radiotherapy

Postoperative chemoradiotherapy (CRT) is widely recommended for patients with head and neck cancer who present with high‐risk factors for recurrence. However, the optimal management of patients with intermediate‐risk factors remains uncertain, and further studies are needed to establish standardized postoperative treatment strategies for this subgroup. Previous studies have demonstrated the efficacy of PORT in reducing recurrence in advanced T‐stage disease [14], and our findings highlight its critical role in minimizing recurrence within the masticator space for contact‐type tumors, even with negative surgical margins. This suggests that microscopic residual disease may persist in the masticator space, contributing to local recurrence despite adequate surgical resection. For intermediate‐risk OSCC, optimizing PORT parameters, including radiation dose and delivery technique, is essential [15]. Advanced techniques such as intensity‐modulated radiotherapy (IMRT) offer precise targeting of complex anatomical regions like the masticator space while minimizing exposure to surrounding normal tissues [16]. IMRT can improve local control and reduce recurrence, particularly when tumors are close to critical structures. These findings emphasize the need for prospective studies to establish tailored treatment protocols for intermediate‐risk patients, incorporating advanced radiotherapy techniques to optimize outcomes while balancing efficacy and minimizing treatment‐associated morbidity.

### 4.3 Relevance of Compartmental Surgery

#### 4.3.1 Discussion

The masticator space plays a crucial role in the progression of OSCC and is recognized as a high‐risk site for local recurrence. Among its anatomical structures, the medial pterygoid muscle functions as a critical barrier against tumor expansion. However, once the tumor infiltrates this muscle layer, it exhibits a characteristic pattern of progression, showing rapid vertical infiltration along the muscle fibers while horizontal spread is relatively delayed. This unique tumor progression pattern affects conventional surgical margin settings, underscoring the need for more aggressive surgical interventions. The findings of this study confirm that even tumors in contact with the medial pterygoid muscle carry a high risk of recurrence, suggesting that a more extensive resection should be considered.

Liao et al. proposed a classification of tumors in the masticator space based on an axial plane at the mandibular notch, defining tumors extending above this plane as supra‐mandibular notch T4b (SN–T4b) and those below as infra‐notch T4b (IN–T4b) [8]. Their study demonstrated that IN–T4b tumors had a significantly better prognosis, with a 5‐year overall survival (OS) rate of 55.3% and a disease‐free survival (DFS) rate of 64.7%; whereas SN–T4b tumors showed markedly lower OS (14.3%) and DFS (21.4%). In a subsequent study comparing IN–T4b and pT4a OSCC, the 5‐year OS rates were 62% versus 44%, and DFS rates were 63% versus 55%, with no significant difference between the groups [17]. These findings suggest that IN–T4b tumors should be reclassified as T4a, warranting a revision of the staging system.

Trivedi et al. proposed the concept of compartmental resection, advocating for an en bloc resection of the entire masticator space, irrespective of tumor extent, to ensure negative soft tissue margins and achieve complete tumor removal [18]. Supporting this approach, Pillai et al. reported that tumor invasion into the lateral pterygoid muscle, a defining feature of SN–T4b, was associated with poor prognosis, as the 2‐year DFS decreased from 66% in cases without lateral pterygoid invasion to 43% in those with invasion [9]. Furthermore, a recent meta‐analysis comparing surgically treated IN–T4b and T4a OSCC found no significant differences in OS, disease‐specific survival (DSS), DFS, or local control rates, reinforcing the argument for downstaging IN–T4b tumors to T4a [10].

Compartmental surgery is expected to play an increasingly pivotal role in OSCC treatment. Recent studies have explored the potential benefits of multiportal approaches, particularly the transnasal endoscopic anterior route, which enhances margin control at the level of the pterygo‐maxillary fissure [19, 20]. Additionally, the recently introduced concept of the posterior oral anatomical complex (POAC) has gained attention as a key factor in surgical strategy for posterior OSCC (POSCC) [21, 22]. The POAC includes the posterior mandibular body, mandibular ramus, infratemporal surface of the maxillary body, pterygoid process, temporalis muscle, medial pterygoid muscle, and lateral pterygoid muscle. The lack of clear anatomical boundaries in the POAC allows tumor cells to spread along muscle fibers and adjacent spaces toward the basicranial region [23]. This unique pattern of tumor spread challenges traditional resection margins, making it difficult to achieve complete local control [24].

Traditional compartmental surgery involves resecting an entire predefined anatomical compartment, regardless of tumor extent, to ensure clear margins. The primary goal of this approach is to minimize recurrence risk by removing all potential soft tissue invasion areas and securing tumor‐free margins [18, 25]. However, this technique poses challenges, as extensive resection increases the risk of functional and aesthetic complications, and a standardized resection approach may not accommodate individualized tumor progression patterns.

In contrast, Anatomical Unit Resection Surgery (AURS) is designed to resect specific anatomical units or subunits based on tumor location and invasion pattern. It is particularly applicable to POSCC with POAC involvement, offering a more tailored surgical strategy compared to compartmental resection. Unlike compartmental surgery, which removes the entire compartment indiscriminately, AURS ensures the complete removal of critical attachment sites such as the internal and external pterygoid plates, maxillary tuberosity, and associated musculature, optimizing the balance between oncologic control and functional preservation. Furthermore, AURS allows individualized surgical planning based on factors such as the need for coronoid process preservation, the extent of mandibular body resection, the necessity for temporalis muscle resection, the degree of maxillary bone removal, and the requirement for facial skin excision.

The clinical outcomes of AURS have demonstrated significant advantages over conventional approaches. Wu et al. reported that the 5‐year OS and DFS rates for the AURS group were 62.5%, compared to 37.5% and 34.3%, respectively, for the conventional surgery group, indicating a clear survival benefit [21]. Furthermore, Ren et al. suggested that for locally advanced OSCC (LA‐OSCC), optimal outcomes can be achieved with surgery alone if appropriate surgical concepts and radical excision are applied, raising questions about the necessity of postoperative adjuvant therapy [26]. Despite the potential benefits of compartmental surgery, particularly for contact‐type OSCC, no established criteria currently exist to guide the decision to employ such an aggressive surgical approach. The lack of specific prognostic or anatomical indicators complicates decision‐making, leaving surgeons to rely on individual judgment rather than standardized guidelines.

Future research should focus on establishing evidence‐based criteria for compartmental surgery indications, defining optimal resection margins for the masticator space, and balancing oncologic control with functional outcomes. By refining surgical strategies and identifying reliable prognostic markers, treatment planning can be optimized, leading to improved long‐term outcomes for patients with advanced OSCC.

## 5 Study Limitations

This study had several limitations. Firstly, as a single‐center, retrospective analysis with a small sample size, the results may have been influenced by selection bias. Secondly, critical factors such as smoking history, alcohol consumption, and comorbidities were not included in the analysis, which could have affected treatment outcomes. Finally, as a retrospective study, the depth of invasion for TNM classification (UICC 8th edition) was assessed using histopathological specimens, which might have differed from clinical assessments.

## 6 Conclusion

In summary, the results highlighted the importance of addressing lymphatic invasion and the role of PORT in managing contact‐type OSCC with a high recurrence risk in the masticator space. Compartmental surgery, tailored to the extent of tumor invasion and supplemented with adjuvant therapy, may provide the best opportunity for long‐term disease control. These findings establish a foundation for future research aimed at refining treatment strategies and improving outcomes for patients with advanced OSCC.

## Author Contributions

**Nanako Ito:** conception and design of study, analysis and interpretation of data, drafting and critically revising work, and final approval of the manuscript. **Fumitaka Obayashi:** analysis and interpretation of data, critically revising work, final approval of the manuscript, and agreement to be accountable for all aspects of the work. **Mirai Higaki**, **Atsuko Hamada, Tomoaki Shintani**, and **Sachiko Yamasaki:** interpretation of data, critically revising work, final approval of the manuscript, and agreement to be accountable for all aspects of the work. **Toshinori Ando:** interpretation of data, final approval of the manuscript, and agreement to be accountable for all aspects of the work. **Koichi Koizumi:** interpretation of data, critically revising work, final approval of the manuscript, and agreement to be accountable for all aspects of the work. **Souichi Yanamoto:** conception and design of study, analysis and interpretation of data, critically revising work, final approval of the manuscript, and agreement to be accountable for all aspects of the work.

## Disclosure

*Declaration of Generative AI*: We did not use any generative artificial intelligence (AI) and AI‐assisted technologies.

*Permission to Reproduce Material From Other Sources*: No previously published material was reproduced in this study.

## Ethics Statement

Ethical approval was obtained from the Institutional Review Board of Hiroshima University Hospital (approval number: epidemiology‐2023‐0025). This study conformed to the principles of the Declaration of Helsinki.

## Conflicts of Interest

The authors declare no conflicts of interest.

## Data Availability

The data that support the findings of this study are available from the corresponding author upon reasonable request.
